# Supplementary material for: Mouse-Geneformer: A deep learning model for mouse single-cell transcriptome and its cross-species utility
Source: PLoS Genet. 2025 Mar 19;21(3):e1011420. doi: 10.1371/journal.pgen.1011420 (PMC11964219; doi:10.1371/journal.pgen.1011420)
Supplement: Attachment — Submitted filename: Response_to_Reviewers.250113b.pdf [file pgen.1011420.s004.pdf]

Response to Reviewers

Manuscript Number: PGENETICS-D-24-01035R1

Mouse-Geneformer: A Deep Learning Model for Mouse Single-Cell Transcriptome and Its Cross-Species Utility
By Keita Ito, Tsubasa Hirakawa, Shuji Shigenobu, Hironobu Fujiyoshi, Takayoshi Yamashita

We would like to express our profound gratitude for the professional advice and constructive comments given by the reviewers on this manuscript. In response, we carefully considered all the comments and revised the manuscript accordingly. In our view, these revisions have led to substantial improvements in the quality and clarity of the manuscript.

Before addressing each comment individually, we would like to provide an overview of the major revisions made to the manuscript, in addition to the many minor edits for overall improvement:

1) **Inclusion of cell type classification analysis using scGPT**: All three reviewers suggested including a comparison with scGPT. As scGPT is a relatively new tool that was not available at the time of our initial manuscript preparation, it was not included in our original analysis. Recognizing its strong performance and growing importance in the field, we have now incorporated scGPT in our comparisons. It is worth noting that scGPT is trained on human data and cannot directly process mouse data. To overcome this limitation, we applied the homology-based gene name conversion method introduced in our study. The successful application of this method further underscores the versatility and effectiveness of the cross-species strategy proposed in our paper.
2) **Analysis of mouse RNA-seq data using human-Geneformer**: In response to a suggestion from a reviewer, we conducted a new experiment analyzing mouse RNA-seq data using human-Geneformer. This experiment represents a reverse cross-species application to complement our original analysis, where we analyzed human data using mouse-Geneformer. The inclusion of this analysis strengthens the manuscript by demonstrating the bidirectional applicability of the cross-species strategy.
3) **Expanded discussion on the cross-species strategy**: As a result of the new analyses described in points 1 and 2 above, we have expanded the description and discussion of the cross-species strategy. This expansion includes two new sections in the Results section: **"Mouse Cell Type Classification Using Human Geneformer"** and **"Cross-Species Approach Using Human-Based Omics Models and Comparison with Mouse-Geneformer"**. Additionally, we have significantly enhanced the last two paragraphs of the Discussion section to provide a more comprehensive interpretation of our findings on cross-species applications.
4) **Improved evaluation metrics for cell type classification**: In our original manuscript, we reported only accuracy as an evaluation metric for cell type classification. In the revised version, we have calculated additional metrics, including precision, recall, and F1 score. All relevant tables have been updated to include accuracy and F1 score, with F1 score representing the harmonic mean of precision and recall.

The point-by-point response

Reviewer Comments:

Notes on the format in this response letter

- black: reviewers' comments
- blue: our answers
- *italic: texts quoted from manuscript*

Reviewer #1

Geneformer is a transformer-based deep learning model designed for single-cell RNA-seq analysis, focusing on learning gene-gene interactions to enable tasks like cell-type classification and gene perturbation prediction. The paper developed a mouse-specific GeneFormer by pretraining Geneformer on a constructed mouse dataset. The architecture and strategy are the same as those that have been used in GeneFormer.

(1-0)

It seems nowadays there's a trend in developing foundation models in scRNA-seq data, such as Geneformer and scGPT. And the authors' endeavor to develop a mouse single-cell foundation model is valuable.

Ans) We deeply appreciate the reviewer's recognition of the significance of our work.

I have the following two major comments and a few minor ones.

(1-1)
1. The authors constructed a large-scale dataset of mouse single-cell RNA-seq data i.e., mouse-Genecorpus-20M, derived from healthy mice. Would it be good to include diseased mouse data in the pre-training? From Fig 2, the training data seem unbalanced in terms of ST platform and organ, etc. What would be the author's comment on this?

Ans) We appreciate the reviewer's thoughtful suggestion. In this study, we focused on constructing a dataset derived exclusively from healthy mice to establish a reference genetic architecture. Our primary concern with including data from diseased mice lies in the potential restructuring of gene networks caused by high mutation loads, which could introduce confounding effects. This approach aligns with the methodology of the original human Geneformer, which also excluded data from cells with mutational alterations.

That said, we recognize the potential benefits of incorporating data from diseased mice, such as increased data volume and diversity, which could enhance model performance. We find this to be an intriguing direction for future exploration and plan to address it as part of subsequent work.

(1-2)
2. The authors demonstrated the advantage of mouse-Geneformer on cell type classification and perturbation experiments. I think to make this a potentially widely used foundation model in mice, the author may demonstrate its potential and advantage of it more comprehensively. For example, for mouse single-cell analysis, similar to GeneFormer, it may be helpful to include more tasks.

Ans) We thank the reviewer for this valuable suggestion. In this manuscript, we focused on two key categories of tasks: cell type classification and in silico perturbation experiments. These were prioritized because they represent some of the most commonly performed tasks in single-cell RNA-seq analysis of mice and are highly relevant to the needs of the mouse research community.

We agree that demonstrating the utility of mouse-Geneformer across a broader range of tasks would further highlight its potential as a widely applicable foundation model. For instance, tasks such as chromatin dynamics prediction and network dynamics prediction, as explored in the original human Geneformer study, are promising directions. Although these tasks were beyond the scope of this manuscript, we are keen to explore them in future work.

Importantly, we have made our mouse-Geneformer models publicly available. We hope this will enable researchers to evaluate its performance on other tasks and uncover new applications, further demonstrating its versatility and utility.

Minor comments:
(1-3)

When evaluating the cell type classification, would it be suitable to include scGPT?

Ans) We thank the reviewer for the insightful suggestion. In this revision, we have incorporated new experiments to compare Mouse-Geneformer with scGPT. As scGPT is a relatively new tool that was not available when we initially prepared our manuscript, we had not included it in our original analysis. However, recognizing its strong performance and growing popularity in the field, we have now included scGPT in our comparisons.

Specifically, we conducted cell type classification tasks across 12 scRNA-seq datasets, and the results are presented in the newly added section, *“Cross-Species Approach Using Human-Based Omics Models and Comparison with Mouse-Geneformer.”* Please note that scGPT is trained on human omics data and cannot directly process mouse data. To address this issue, we applied the homology-based gene name conversion method proposed in our study. The successful performance of scGPT using this conversion further highlights the effectiveness of the cross-species strategy we introduced.

(1-4)

Fig. 2, maybe a pie chart?

Ans) We appreciate the reviewer's suggestion regarding the use of a pie chart for Fig. 2. While pie charts can be effective for quickly visualizing proportions and are often self-explanatory with minimal labeling (e.g., Hill, 2024: Are Pie Charts Evil?), they are generally not recommended for scientific data presentation due to potential difficulties in interpretation and the risk of misrepresenting data (e.g., Healy, 2018: Data Visualization). In this manuscript, we chose to use bar charts for Fig. 2 because the figure includes numerous distinct labels. Displaying such information in a pie chart would make it challenging to read and interpret accurately. We believe the current bar chart format provides a clearer and more precise representation of the data.

(1-5)
Fig. 4, maybe adjust the size of the dots for better visualization?

Ans) We agree that the UMAP plots of Fig. 4 in the original manuscript required improvement for better visualization. Upon review, we also recognized that Fig. 5 could benefit from similar adjustments. In response, we have revised Fig. 4 and Fig. 5 to enhance the clarity of data point distribution. Specifically, we reduced the size of the dots in the UMAP plots, which allows for a more detailed and refined visualization of the data distribution.

//

Reviewer #2

In this manuscript, the authors collected 20 M single-cell RNA sequencing (scRNA-seq) data from mice and trained a model called mouse-Geneformer, based on the existing Geneformer architecture. The rationale for training this model stems from the widespread use of mice as model organisms in biomedical research. Additionally, the manuscript explores the potential of cross-species applications, demonstrating the promise of mouse-Geneformer for cross-species analysis. Interestingly, it achieves cell type classification accuracies comparable to those of the original human Geneformer model. Their results suggest that mouse-Geneformer can be a valuable tool not only for mouse biology but also for cross-species transcriptomic analysis, potentially benefiting research on non-model organisms and human data inaccessible due to ethical constraints.

With merits being said, despite the large scale of the dataset, the study does not introduce any novel models or algorithms from a methodological perspective, but instead simply replaces the pretraining dataset of the original Geneformer model. This may weaken the manuscript's overall innovation. Furthermore, the evaluation of model performance and cross-species analysis is not sufficiently robust, failing to effectively demonstrate the significance of mouse-Geneformer and its advantages over existing methods. Specific comments are given as follows.

(2-0)

Ans) We thank the reviewer for their constructive feedback. As the reviewer correctly noted, our mouse-Geneformer is based on the existing human-Geneformer architecture, and this study does not introduce novel algorithms. We acknowledge this limitation and wish to clarify that the primary goal of this study was not to propose new methodologies but rather to demonstrate the robustness and applicability of the Geneformer architecture when applied to a large-scale mouse dataset. This serves as a foundational step for future developments. In fact, we are currently working on developing new tools that incorporate more advanced architectures such as Mamba, which we plan to publish separately in the near future. This work will build upon the insights gained from the current study.

Regarding the comment that "the evaluation of model performance and cross-species analysis is not sufficiently robust," we have carefully revised the manuscript to address this concern. Specific changes and improvements are detailed in our responses below.

Major

(2-1)
1. Comparison with the original human Geneformer: Has the authors attempted to analyze mouse data using the original Geneformer by fine-tuning with mouse data? If so, did fine-tuning or direct use of the original Geneformer produce results similar to those of mouse-Geneformer in downstream mouse tasks? If comparable results can be achieved, the necessity of developing a mouse-specific version (mouse-Geneformer) might need further justification. Even if mouse-Geneformer outperforms the original Geneformer in certain aspects, the authors should provide a detailed performance comparison between the two models to substantiate the advantages of developing a mouse-specific version.

Ans)

We appreciate the reviewer’s valuable suggestion. In response, we have conducted additional analyses using the original human Geneformer to evaluate its performance on mouse data. For this purpose, we applied homology-based gene name conversion, similar to the approach used for mouse-Geneformer when analyzing human data. The results of this analysis are presented in the newly added section, “Mouse Cell Type Classification Using Human Geneformer” [p.99, L123] in the Results, along with the new Table 5.

Our findings demonstrate that the human Geneformer successfully classified mouse cell types based on mouse transcriptome data across all cases, achieving performance levels comparable to those of mouse-Geneformer. As the reviewer pointed out, the strong performance of human Geneformer in mouse data raises the question of whether developing a mouse-specific version is necessary. We addressed this issue in the same section by examining the comparative results in detail.

Notably, we observed that tasks involving cell type classification with a larger number of classes exhibited reduced performance when using the cross-species application of Geneformer. This suggests that more complex tasks require native transcriptome data for high-resolution classification, whereas simpler tasks, such as those involving fewer cell types, can be effectively analyzed using a cross-species approach. We believe these insights underscore the importance of mouse-Geneformer for advancing research on tasks requiring higher resolution and task-specific optimization.

(2-2)

2. Limitations of cross-species homology-based gene conversion: While gene orthology conversion strategies can be useful in cross-species studies, gene homology alone cannot fully account for biological functional differences between species. Even when genes are homologous, their regulatory mechanisms, spatiotemporal expression patterns, and involvement in biological pathways can vary significantly across species. These differences may contribute to the model's inconsistent performance in cross-species applications (e.g., as noted in the differing COVID-19 results). The authors should discuss which types of analyses this orthology conversion strategy is best suited for. Is it applicable to all genes, particularly those with divergent biological functions across species? What is the practical applicability of this strategy in real biological research? Without deeper discussion of these questions, the reliability of cross-species application of the model may be called into question.

We thank the reviewer for highlighting this critical issue. As the cross-species strategy using homology-based gene conversion is a key message of our paper, we fully agree that a deeper discussion on its applicability and limitations is necessary to clarify the reliability and practical use of this approach. In this revision, we have significantly expanded the Discussion section to address these concerns in detail. Specifically, we enhanced two paragraphs that discuss both the advantages and limitations of the cross-species strategy, including its applicability to different types of genes, biological and evolutionary contexts. These paragraphs can be found in the Discussion section, corresponding to [L739-784].

We demonstrated that a cross-species approach using homology-based gene name conversion is effective in three scenarios: (1) analyzing human data with mouse-Geneformer, (2) analyzing mouse data with human-Geneformer, and (3) analyzing mouse data with scGPT, a model built upon human transcriptome data. Despite these successful applications, it is important to acknowledge the limitations of the homology-based cross-species strategy, as gene homology alone cannot fully account for the functional and regulatory differences between species. Even when genes are homologous, their regulatory mechanisms, spatiotemporal expression patterns, and roles in biological pathways can vary significantly across species. In fact, we observed that mouse-Geneformer failed to fully predict the outcome of perturbation experiments on human COVID-19 blood cell data, whereas the original human Geneformer predicted more accurately. Note that, SARS-CoV-2, the coronavirus responsible for COVID-19, does not naturally infect wild-type laboratory mice, highlighting the species-specific differences in viral susceptibility. On the other hand, we predict that housekeeping metabolic processes involving highly conserved genes can be effectively analyzed across species. It is also possible that lineage-specific

phenotypes are influenced by complex genetic networks that integrate both conserved core pathways and species-specific regulatory components. In such cases, even lineage-specific traits may be partially analyzed using a cross-species approach, with conserved genetic elements contributing to the predictive accuracy of the model. Additionally, we observed that cell type classification tasks with a higher number of classes showed reduced performance in cross-species applications of Geneformer. This suggests that more complex tasks require native transcriptome data for high-resolution classification, as cross-species models may lack the species-specific information necessary to achieve the same level of accuracy. Further research is needed to explore the full potential and limitations of cross-species applications of Geneformer models, particularly in understanding how conserved and species-specific gene networks interact to influence biological processes across different organisms.

The cross-species application of Geneformer holds great potential for the analysis of non-model organisms, for which it is difficult, costly, and technically challenging to obtain sufficient single-cell transcriptome data to construct species-specific Geneformer models. However, since we investigated only a single combination of cross-species application between human and mouse, it remains unclear how closely related species can be analyzed with Geneformer models in a cross-species manner. Future research should explore the utility of cross-species applications across various evolutionary distances, such as within or across families, orders, or even phyla. It is reasonable to predict that the greater the evolutionary distance between species, the more challenging these applications will become. This is likely due to several factors, including reduced conservation of genetic regulatory networks, diminished functional conservation of homologous genes, and technical difficulties in accurately identifying orthologs across distantly related species. The success of such applications may also depend on the traits or genes of interest. We predict that housekeeping metabolic processes involving conserved genes can be analyzed across species, whereas lineage-specific traits may not be as effectively analyzed. Importantly, the cross-species approach is not limited to Geneformer but is a versatile strategy that can be applied to other large-scale omics models. In fact, we demonstrated the successful use of human-based scGPT for mouse data, further supporting the feasibility of this approach. Looking ahead, it is inevitable that more advanced algorithms and tools will be developed to model large-scale omics data for human and mouse. These tools will likely be applicable to other species of interest, enabling researchers to leverage existing resources in a cross-species manner to address a wide range of biological questions.

(2-3)
3. Cross-species experiment clarity. While the ortholog-based gene conversion method showed near-perfect accuracy (99.97%) for human cell type classification, this result is unexpectedly high. It would be insightful for the authors to compare this method with other baseline models, such as logistic regression, scVI, or foundation models like scBERT and scGPT, to confirm if the high accuracy is genuinely due to the ortholog conversion or if the task itself is particularly easy.

Ans) Upon further investigation, we found that the exceptionally high accuracy (99.97%) is primarily due to the relative simplicity of the task. To support this conclusion, we conducted additional analyses using several methods, including Mouse-Geneformer (zero-shot), Human-Geneformer (zero-shot), and scGPT. The results are summarized in the updated Table 4. The results showed that all methods achieved very high accuracy for cell classification in the cerebral cortex, confirming the simplicity of the task. Notably, even Mouse-Geneformer (zero-shot), without any fine-tuning on human data, demonstrated strong performance, achieving an accuracy of 89.17%, which indicates the easiness of this classification task.

(2-4)
4. Inconsistent datasets in similar tasks: In the sections 'Cell type classification using the mouse-Geneformer and other methods' and 'Cell type classification using mouse-Geneformer with and without prior learning', the authors use different datasets but do not explain why different datasets are used for the same task type. Specifically, in Table 2, the mouse-Geneformer achieves an accuracy greater than 0.9, whereas in Table 3, the accuracy on two different datasets hovers around 0.7. How do other methods perform on these two datasets in comparison? The lack of consistent dataset usage for similar tasks raises questions about the validity of comparisons across experiments.

Ans)
We sincerely thank the reviewer for this valuable feedback. We fully agree that consistent dataset usage is crucial for making valid comparisons across similar tasks. In this revision, we addressed this concern by conducting additional experiments to ensure that the section "Cell Type Classification Using Mouse-Geneformer and Other Methods" includes the same dataset used in 'Cell type classification using mouse-Geneformer with and without prior learning', allowing for a direct comparison between algorithms. Specifically, we analyzed 12 scRNA-seq datasets in total, spanning various tissues, and the results are now reflected in the updated Table 2.

Additionally, to enhance the logical flow of the manuscript, we revised the order of these two sections. We now present the section *"Cell Type Classification Using Mouse-Geneformer with and Without Prior Learning"* first, as it demonstrates the efficiency of pretraining—a key foundation for the subsequent experiments including "Cell Type Classification Using Mouse-Geneformer and Other Methods", which predominantly use pretrained models. We believe this reordering should improve the overall readability and coherence of the manuscript.

(2-5)
5. Limited model comparisons and evaluation metrics: In the 'Cell type classification using the mouse-Geneformer and other methods' task, the authors compare mouse-Geneformer with only two other methods, scDeepSort and scVAE, which seems limited given the broad scope of the field. Additionally, accuracy is the sole evaluation metric presented, which may not sufficiently capture model performance, especially in the presence of class imbalances. Accuracy alone may overlook how the model performs on minority cell types, which are often critical in scRNA-seq analyses. Furthermore, since mouse-Geneformer was fine-tuned on the dataset, what are the results in a zero-shot setting?

Ans）We thank the reviewer for the insightful suggestions. In response, we have added a new comparison experiment using scGPT for cell type classification. The results are presented in the newly added section, “Cross-Species Approach Using Human-Based Omics Models and Comparison with Mouse-Geneformer” [L673]. Please note that since scGPT is based on human omics data, it cannot directly process mouse data. Therefore, we applied the homology-based

gene name conversion method proposed in our study. As detailed in the manuscript, scGPT demonstrated strong performance with this conversion, further highlighting the effectiveness and usability of the cross-species strategy we proposed.

Regarding the comment that *"accuracy is the sole evaluation metric presented, which may not sufficiently capture model performance, especially in the presence of class imbalances,"* we fully agree. In classification tasks, metrics such as precision, recall, and F1 score are commonly used alongside accuracy to provide a more comprehensive evaluation of model performance. In this revision, we chose to include the F1 score as an additional metric, as it represents the harmonic mean of precision and recall, balancing both metrics and providing a more reliable assessment in the presence of imbalanced classes. We have updated all relevant tables in the manuscript to report both accuracy and F1 score.

Lastly, in response to the reviewer's request for zero-shot results, we have included the performance of Mouse-Geneformer in a zero-shot setting. These results are presented in the largely updated Table 5. We believe that the inclusion of these additional experiments and metrics provides a more thorough and balanced evaluation of our model's performance.

Minor:

(2-6)
1. Add more visualizations to show the results. It's recommended to include additional figures in the paper to better demonstrate key findings, especially for classification and perturbation results.

Ans) We thank the reviewer for this suggestion. While we did not add entirely new figures in this revision, we have incorporated additional experiments on classification tasks in response to feedback from all reviewers. These new experiments are presented in the form of updated tables, which we believe contribute to a clearer and more comprehensive demonstration of our key findings.

//

Reviewer #3

Reviewer #3: Major concerns.

(3-1)
1. scGPT is an alternative to geneformer. could the authors give some brief discussions on the difference between scGPT and geneformer, and why to build their model based on geneformer

instead of scGPT?

Ans）We thank the reviewer for raising this important point. In this revision, we have included new experiments comparing Mouse-Geneformer with scGPT. It is worth noting that scGPT is a relatively new tool that was not yet available when we initially prepared our manuscript. However, recognizing its excellent performance and growing popularity, we decided to include scGPT in our comparisons. Specifically, we conducted cell type classification tasks across 12 scRNA-seq datasets. The results are presented in the newly added section, “Cross-Species Approach Using Human-Based Omics Models and Comparison with Mouse-Geneformer.” Since scGPT is trained on human omics data, it cannot directly process mouse data. To address this, we applied the homology-based gene name conversion method proposed in our study. As detailed in the manuscript [L673], scGPT demonstrated strong performance with this conversion, further emphasizing the effectiveness of our cross-species strategy.

(3-2)
2. How are batch effect (e.g., platform) being handled in both training and inference stages?

Ans) We thank the reviewer for raising this important point. Batch effects are handled using the Rank Value Encoding method, as developed in the original human Geneformer. In this method, the transcriptome of each single cell is represented by ranking genes based on their expression within that cell, normalized by their expression across the entire Genecorpus. As discussed in the original Geneformer paper (Theodoris et al., 2023 Nature; Section titled "Context Awareness and Batch Integration"), this Rank Value Encoding approach is robust to batch-dependent technical artifacts, effectively mitigating batch effects. Our Mouse-Geneformer employs the same algorithm, inheriting the same robustness to batch effects. Therefore, we expect our model to maintain this robustness during both the training and inference stages.

(3-3)
3. The genetic difference and transcriptional difference of different mouse strains can be huge. How are they handeled in the model.

Ans) In this study, our primary focus was to construct a reference genetic architecture based on data from healthy mice. While we excluded mutant mice and cell lines, we did not specifically account for differences among mouse strains. So, we here investigated the mouse strain diversity in the dataset. We noticed that most large-scale scRNA-seq database do not provide comprehensive information on the mouse strains used. However, based on our survey of a subset of datasets from PanglaoDB (https://panglaodb.se/) along with corresponding SRA

database at NCBI, we found that the majority of the data were derived from C57BL/6 mice, the most commonly used inbred strain in laboratory research (see the table below). Therefore, we believe that the genetic diversity within the mouse-Genecorpus20M dataset is relatively limited, and the resulting transcriptional differences should be minimal.

SRA	SRC	Tissue/Site	strain
SRA644036	SRS2808714	Cervical and lumbar spinal cord	mix of C57BL/6J and CB6F1/J
SRA670243	SRS3078084	Ventral midbrain	CD-1
SRA689041	SRS3166675	Colon	C57BL6
SRA599311	SRS2431648	Mesenteric	C57BL/6N
SRA599311	SRS2431649	Mesenteric	C57BL/6N
SRA571721	SRS2259442	Bone marrow stem cells	C57BL/6 Spp1-/-
SRA571721	SRS2259441	Bone marrow stem cells	C57BL/6
SRA667466	SRS3060061	Enteric nervous system	Wnt1Cre-R26Rtomato-C57BL/6J
SRA667466	SRS3060069	Amygdala	CD-1
SRA526435	SRS1915256	Cortex, hippocampus and subventricular zone	C57BL/6
SRA526435	SRS1915253	Cortex, hippocampus and subventricular zone	C57BL/6
SRA526435	SRS1915247	Cortex, hippocampus and subventricular zone	C57BL/6
SRA526435	SRS1915240	Cortex, hippocampus and subventricular zone	C57BL/6
SRA550869	SRS2747908	Aortic leukocytes	C57BL/6J
SRA550869	SRS2747909	Aortic leukocytes	C57BL/6J
SRA550869	SRS2093772	Aortic leukocytes	C57BL/6J
SRA578634	SRS2302660	Subventricular zone	C57/Bl6(3/4) x 129S1SvlmJ(1/4)
SRA578634	SRS2302659	Subventricular zone	C57/Bl6(3/4) x 129S1SvlmJ(1/4)
SRA578634	SRS2302658	Subventricular zone	C57/Bl6(3/4) x 129S1SvlmJ(1/4)
SRA688150	SRS3161265	Lung	C57BL6
SRA688150	SRS3161263	Lung	C57BL6
SRA688150	SRS3161264	Lung	C57BL6

SRA688150	SRS3161262	Lung	C57BL6
SRA866994	SRS4545972	Choroid plexus	CD64Cre/+ x Irf8fl/fl
SRA866994	SRS4545968	Choroid plexus	C57BL/6JRj
SRA866994	SRS4545965	Dura mater	C57BL/6JRj
SRA866994	SRS4545961	Brain	C57BL/6JRj

It is also worth noting that the original human Geneformer was trained on scRNA-seq data from a much broader range of human individuals with significant genetic and transcriptomic diversity. Despite this, the Geneformer architecture demonstrated robust and reliable performance across a wide variety of downstream tasks. Based on this precedent, we expect that the mouse-Geneformer, which is built using a more genetically homogeneous dataset, would similarly exhibit robustness.

(3-4)
4. for "Cell type classification using the mouse-Geneformer and other methods", "The mouse data used for comparison comprised single-cell data from nine organs of mice: tongue [25], thymus [25], mammary gland [25], large intestine [25], limb muscle [25], spleen [25], heart [25], brain [25], and kidney [25]." Are these data part of the 20M pre-training data? If not, is there a way to evaluate how similar these data to the training data? the data leaking is always a concern for deep learning. Similar question of data similarity also applies to the "Cell type classification using mouse-Geneformer with and without prior learning".

Ans）We thank the reviewer for rainsing the concerns regarding potential data leakage. We agree that ensuring the independence of the evaluation data is a critical issue in deep learning. First, we would like to clarify that the mouse-Genecorpus-20M dataset does not include the scRNA-seq data used for evaluation in the studies on “Cell type classification using mouse-Geneformer and other methods" and “Cell type classification using mouse-Geneformer with and without prior learning.“ The evaluation datasets were entirely independent of the pretraining data.

Next, we assessed the similarity between mouse-Genecorpus-20M and these evaluation datasets by comparing the features of the pretraining and evaluation data using Euclidean distance. The results showed that while there are pretraining data points with Euclidean distances close to 0, there are no cases where the distance is exactly 0. In cases of data leakage, the Euclidean distance would be 0, indicating complete feature matching. However, no such complete matches were observed in our analysis.

The presence of data points with Euclidean distances close to 0 is likely due to the fact that both the evaluation and pretraining data originate from the same organs or similar cell types. This

supports the conclusion that the evaluation data remain independent and that the model has learned generalized patterns rather than depending on specific data.

To clarify the absence of data leakage in the manuscript, we added the sentence like "… were not included in the mouse-Genecorpus-20M. To confirm the integrity of the data, we compared features using Euclidean distance, and the results indicated that no data was completely zero, confirming the absence of data leakage." were added to the corresponding locations in Materials and Methods.

(3-5)
5. "The mouse-Geneformer was then fine-tuned for disease classification tasks, ensuring that the disease classification accuracy of the test data exceeds 90%.". What kind of disease classification task? the disease should be labeled at the level of mice? but the classification is done on the level of cells?

Ans) The original version did not provide sufficient explanation on the procedure. We have added details in "In silico perturbation experiments" in Materials and Methods as follows:

[L262] The in silico gene manipulation experiment using Mouse-Geneformer applied perturbations to each gene to identify those that contribute significantly to the disease. The Mouse-Geneformer in silico perturbation experiment first normalized the gene expression data and then applied Rank Value Encoding to rank the expression of each gene, creating a ranked gene table. Then, Mouse-Geneformer was fine-tuned for disease classification tasks, ensuring that the disease classification accuracy on the test data exceeds 90%.

[L284] Disease labels were assigned at the organ level: cells from diseased organs were labeled as diseased, while cells from normal organs were labeled as normal. The cells were then classified according to these labels.

(3-6A)
6. "When deleting a gene, the gene was removed from the dataset, and the rank values of other genes were increased. Conversely, when activating a gene, the rank value of that gene was increased, and the rank values of other genes were decreased. "

First, these statements are confusing. "When deleting a gene, the gene was removed from the dataset". Is it really removed or just set its expression to 0? "the rank values of other genes were increased", should it be "the rank values of the genes with lower ranks were increased"? For the part "when activating a gene, the rank value of that gene was increased, and the rank

values of other genes were decreased." How much does the rank value of that gene was increased? again, the rank values of subset of genes rather than all the genes were decreased?

Ans) When deleting a gene, instead of setting its expression value to 0, we completely removed it from the dataset. Subsequently, the rank values of genes with lower ranks than the deleted gene were each increased by one. Conversely, when activating a gene, it was repositioned to have a rank value of 1, and the rank values of genes ranked higher than the activated gene were each decreased by one.

To clarify these procedures, we revised the text of "In silico perturbation experiments section" in Materials and Methods as follows:

[L268] For "virtual" gene deletion in silico, rather than setting its expression value to 0, we completely removed the gene from the dataset. The rank values of genes with lower ranks than the deleted gene were each increased by one. Conversely, for "virtual" activating a gene in silico, the target gene was repositioned to have a rank value of 1, and the rank values of genes ranked higher than the activated gene were each decreased by one. Cosine similarity was used to quantify the distances between the perturbed cell state and a specific cell state.

(3-6B)
Second are these the procedure to simulate perturbed data? then it is very unrealistic to assume only one gene is perturbed without any effect on other genes?

Ans) In the in silico perturbation experiments, we aim to mimic gene knockout experiments in the laboratory, where a specific target gene is disrupted. Typically, such experiments involve perturbation of a single gene, and our approach reflects this. In our model, removing a single gene from the gene list using the Rank Value Encoding method corresponds to simulating this gene knockout scenario.

As the reviewer rightly pointed out, deleting a single gene is expected to affect the expression of many other genes due to downstream regulatory interactions. These secondary effects are precisely what the Geneformer models are designed to predict, capturing how the perturbation of one gene influences broader gene expression networks. Therefore, while we simulate the perturbation of a single gene, the model inherently accounts for the cascade of transcriptional changes that follow.

There are also some minor issues.

(3-7a)
1. "3) Cells with fewer than seven detected genes per cell were removed. 4) Cells with more than 20,000 total gene expression levels were excluded. " Is this too extreme to have only 8-100 genes expressed per cell? "Cells with more than 20,000 total gene expression levels were

excluded" means more than 20,000 detected genes?

Ans) We apologize for the lack of clarity in the original manuscript, which may have caused misunderstanding regarding the filtering criteria. We have revised the description in the Materials and Methods section to provide a more accurate and detailed explanation of the filtering conditions. The updated text reads as follows:

[L167] The specific filtering conditions applied were as follows: 1) For each dataset, cells with total gene expression levels more than three standard deviations from the mean were excluded. 2) Cells with mitochondrial RNA expression levels more than three standard deviations from the mean were excluded. 3) Cells with fewer than seven detected genes per cell were removed. 4) Cells where the sum of the expression levels of all genes per cell exceeded 20,000 were excluded, as excessively high expression levels may indicate artifacts such as doublets or multiplets.

(3-8)
2. caption of table 1 "Eval 2 is its using" -> "Eval 2 uses"

Ans) We thank the reviewer for pointing out the grammatical error in the caption of Table 1. We have corrected it accordingly. Upon further review, we identified additional grammatical issues in the caption and have revised it comprehensively. The updated caption is as follows:

*[L195-200] *Each column represents the parameters used in the the following experiments: Pretrain – pretraining of the mouse-Geneformer; Eval 1 – fine-tuning for cell type classification using mouse-Geneformer and conventional methods; Eval 2 – cell type classification using mouse-Geneformer with and without prior learning; Eval 3 – in silico perturbation experiments using mouse-Geneformer; Eval 4 – cell type classification after gene name conversion between human and mouse. Eval 5 – in silico perturbation experiments after gene name conversion between human and mouse.*

(3-9)
3. "For cell type classification with scDeepSort, a pretrained deep learning model utilizing weighted Graph Neural Networks (GNNs) for single-cell RNA-seq analysis [29], we employed scDeepSort, which consists of a three-layer graph neural..." this sentence need to be rewritten. There are also other places in the paper where the wording or organization of sentence need some edits.

Ans) We thank the reviewer for pointing out the need to revise the sentence. We have rewritten the sentence as follows:

[L233] For cell type classification with scDeepSort, a pretrained deep learning model utilizing weighted Graph Neural Networks (GNNs) for single-cell RNA-seq analysis [29], we employed its three-layer graph neural network architecture [24].

Additionally, we conducted a thorough review of the entire manuscript and corrected several grammatical and structural issues to improve clarity and readability.
