# Supplementary material for: Mouse-Geneformer: A deep learning model for mouse single-cell transcriptome and its cross-species utility
Source: PLoS Genet. 2025 Mar 19;21(3):e1011420. doi: 10.1371/journal.pgen.1011420 (PMC11964219; doi:10.1371/journal.pgen.1011420)
Supplement: Attachment — Submitted filename: Response_to_Reviewers.R2.pdf [file pgen.1011420.s005.pdf]

Response to Reviewers

Manuscript Number: PGENETICS-D-24-01035R1

Mouse-Geneformer: A Deep Learning Model for Mouse Single-Cell Transcriptome and Its Cross-Species Utility
By Keita Ito, Tsubasa Hirakawa, Shuji Shigenobu, Hironobu Fujiyoshi, Takayoshi Yamashita

We appreciate the opportunity to revise our manuscript and are pleased that two of the reviewers found the previous revisions satisfactory. We also sincerely thank Reviewer #3 for their further constructive feedback. In response, we have carefully considered all comments and revised the manuscript accordingly. We believe these revisions have further strengthened the quality and clarity of our work, and we hope the updated manuscript meets the reviewers' expectations.

The point-by-point response

Reviewer Comments:

Notes on the format in this response letter
- black: reviewers' comments
- blue: our answers
- *italic: texts quoted from manuscript*

Reviewer #1

Thank you for addressing/discussing my comments.

Ans) We sincerely appreciate the reviewer's thoughtful feedback and are grateful for the opportunity to improve our manuscript.

Reviewer #2

All comments have been addressed.

Ans) We sincerely appreciate the reviewer's feedback and their recognition of our efforts in revising the manuscript.

Reviewer #3

1. For the question on the genetic difference and transcriptional difference of different mouse strains. The authors answered

"We found that the majority of the data were derived from C57BL/6 mice".

"It is also worth noting that the original human Geneformer was trained on scRNA-seq data from a much broader range of human individuals with significant genetic and transcriptomic diversity. Despite this, the Geneformer architecture demonstrated robust and reliable performance across a wide variety of downstream tasks. Based on this precedent, we expect that the mouse-Geneformer, which is built using a more genetically homogeneous dataset, would similarly exhibit robustness."

The genetic difference between different mouse strains is much larger than the genetic difference between human races. They are comparable to the genetic difference between human and chimpanzees. Therefore, "majority of the data were derived from C57BL/6 mice" is diffrent from all the data were derived from one C57BL/6 mice, and it may not means the model was built using a more genetically homogeneous dataset. I understand it is a lot to ask to trace down the genetic origin of each dataset. At least the authors should acknowledge that this is a potential concern, and the users should keep the mouse strain in mind when using this model.

Ans) We sincerely appreciate the reviewer's insightful comments regarding the genetic and transcriptional differences among mouse strains. We acknowledge that the genetic variation between different mouse strains is significantly greater than that between human populations. As we have reported, the majority of the data used in our model were derived from C57BL/6 mice. However, it does not guarantee that the dataset is entirely homogeneous, as the reviewer pointed out. We agree this view.

Recognizing this as a potential concern, we have revised the manuscript to explicitly state that users should consider the issue of genetic background. We have added the following clarification to the Materials and Methods section:

[L145] *The majority of the datasets were derived from C57BL/6 mice or closely related strains. However, the precise genetic composition of the dataset remains uncertain due to the limited availability of strain annotations in public databases. Given the substantial genetic and transcriptional differences among mouse strains, users should carefully consider these factors when applying this model, particularly in contexts where genetic diversity may influence the results.*

2. (3-6B)
Second are these the procedure to simulate perturbed data? then it is very unrealistic to assume only one gene is perturbed without any effect on other genes?

"Ans) In the in silico perturbation experiments, we aim to mimic gene knockout experiments in the laboratory, where a specific target gene is disrupted. Typically, such experiments involve perturbation of a single gene, and our approach reflects this. In our model, removing a single gene from the gene list using the Rank Value Encoding method corresponds to simulating this

gene knockout scenario."

"As the reviewer rightly pointed out, deleting a single gene is expected to affect the expression of many other genes due to downstream regulatory interactions. These secondary effects are precisely what the Geneformer models are designed to predict, capturing how the perturbation of one gene influences broader gene expression networks. Therefore, while we simulate the perturbation of a single gene, the model inherently accounts for the cascade of transcriptional changes that follow."

Maybe I miss something here. The question I asked was how the author simulated the expression change of other genes? If the expression change of other genes were not simulated, but predicted by Geneformer, how the authors evaluated such prediction?

Ans) We appreciate the reviewer's clarification and the opportunity to provide a more detailed explanation.

In our *in silico* perturbation experiments using Mouse-Geneformer, changes in the expression of other genes affected by the perturbation of a specific gene are not explicitly simulated but rather predicted based on the model's learned gene-gene relationships. In other words, Mouse-Geneformer infers how gene perturbations affect the overall cellular state based on its pretraining on large-scale single-cell transcriptomic data.

To evaluate the predictions, we applied a statistical approach following the methodology used in the original human Geneformer study. Specifically, we assessed the significance of predicted gene perturbation effects using the Wilcoxon rank-sum test (Mann-Whitney U test). The detailed evaluation procedure is as follows:

1. **Baseline Cell State:** The gene expression profiles of reference mouse cells were input into Mouse-Geneformer to compute feature vectors, which represent the gene expression state of each cell. The mean feature vector was then calculated as a reference for comparison.
2. **Perturbed Cell State:** The gene expression profiles of cells with a specific gene perturbation were input into Mouse-Geneformer, generating a new set of feature vectors.
3. **Cosine Similarity Analysis:** The cosine similarity between these perturbed feature vectors and the mean baseline feature vector was computed, yielding the "cosine similarity distribution for specific gene perturbations."
4. **Random Gene Perturbation Control:** As a control, random genes were perturbed in the same dataset, and the corresponding feature vectors were computed. The cosine similarity between these randomly perturbed vectors and the mean baseline feature vector was then calculated, producing the "cosine similarity distribution for random gene perturbations."

5. **Statistical Evaluation:** The Wilcoxon rank-sum test was applied to compare these two cosine similarity distributions. The null hypothesis states that "the effect of perturbing a specific gene on the cell state is equivalent to that of perturbing a random gene." If the p-value is below 0.05, the null hypothesis is rejected, indicating that the specific gene perturbation has a statistically significant impact on the cell state.

6. **Biological Validation:** Finally, the predicted gene set was cross-referenced with known disease-related genes from *in vivo* experimental data to assess the biological relevance of the *in silico* perturbation predictions.

We realized that these methodological details were not sufficiently described in the original manuscript. Therefore, we have now incorporated this information into the **Materials and Methods** section with the following addition:

[L280] *To analyze the effect of genetic perturbations, statistical significance was assessed using the Wilcoxon rank sum test (Mann-Whitney U test), following the approach used in the original human Geneformer study. First, the gene expression table of each reference mouse cell is input into mouse-Geneformer, and an average feature vector representing the gene expression state of the cell was computed. Next, the gene expression table of a cell with a perturbed specific gene was input into mouse-Geneformer to generate a new set of feature vectors. The cosine similarity between these feature vectors and the reference average feature vector was then calculated to obtain the "distribution of cosine similarity under specific gene perturbation." To establish a control, a random gene in the gene table of each perturbed mouse cell was perturbed, and the feature vectors were computed in the same manner. The cosine similarity between this set of feature vectors and the reference average feature vector was then calculated, yielding the "distribution of cosine similarity under random gene perturbation." The Wilcoxon rank-sum test was performed on these two distributions to test the null hypothesis: "The effect of perturbing a specific gene on cell state is comparable to that of perturbing a random gene." If the p-value was below 0.05, the null hypothesis was rejected, indicating that the perturbation of the specific gene has a statistically significant effect on the cell state. This statistical approach allowed for a quantitative assessment of the extent to which perturbation of a specific gene affects cell state compared to random perturbation, thereby enabling the prediction of genes potentially associated with disease.*
